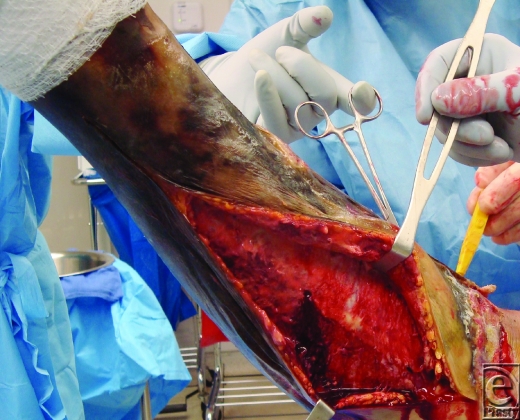# A Crabbing Misadventure

**Published:** 2011-05-13

**Authors:** Hew D.T. Torrance, Alfredo C. Cordova, Ian C. Hoppe

**Affiliations:** ^a^Guy's King's & St Thomas' Hospitals Medical School, London; ^b^The Johns Hopkins Bayview Burn Center, Baltimore, MD; ^c^New Jersey Medical School, Newark

## DESCRIPTION

A 62-year-old diabetic gentleman presented febrile, 38.9°F, with an infected, necrotic left leg. He had previously received 2 weeks of intravenous (IV) antibiotics for cellulitis. His leg was neurovascularly intact with a history significant only for a previous laceration to his leg, prior to a crabbing excursion.

1. **What is the differential diagnosis from Figure 1?**
2. **What is the differential diagnosis now from Figure 2?**
3. **What would be your initial therapy?**
4. **What is the definitive treatment of the wound?**

## DISCUSSION

The initial differential diagnoses include a wide variety of conditions ranging from a chemical burn or an ischemic ulcer to rarer causes such as necrotizing fasciitis, purpura fulminans, pyoderma gangrenosum, erythema induratum, or acute febrile neutrophilic dermatosis.

Figure 2 shows purulent and infected tissue and fascia typical of necrotizing fasciitis. In this case the patient had initially been diagnosed with a *Vibro* bacteremia for which he had been treated with IV antibiotics for a period of 2 weeks. This had not adequately eradicated the infection.

When he was taken to the operating room for debridement of the necrotic left leg, extensive necrotizing fasciitis was discovered. The patient was treated with radical fascial excision and skin grafting.

Necrotizing fasciitis is a rare infection characterized by a widespread necrosis of the subcutaneous tissue and fascia. It is usually described in the lay-press as the product of a flesh-eating bacteria. The bacteria tracks subcutaneously, producing endo- and exotoxins, leading to rapid destruction of the soft tissue.^1^ This results in tissue ischemia and liquefactive necrosis with a mortality rate of 25% to 35%.^2^

It can be subdivided into 3 basic microbial classifications.

Type 1 infections are the most common (55%-75%) and are polymicrobial, usually caused by gram-positive cocci, gram-negative rods, and anaerobes.^3^ It is more commonly seen in the perineal and trunk areas and diagnosed more often in immunocompromised patients.^4^

The second type is a monomicrobial infection, usually caused by group A streptococcus (*Streptococcus pyogenus*). It can also be associated with *Staphylococcus aureus*, producing a toxic shock syndrome. This form of infection tends to occur in healthy, young, nonimmunocompromised patients.^5^

The third and final type is caused by *Vibrio vulnificus*. This infection is usually due to a minor laceration to the skin and exposure to warm seawater,^6^ as typified in our patient.

Aggressive treatment with a number of broad-spectrum IV antibiotics and resuscitation to achieve fluid, electrolyte, and hemodynamic stability should be commenced as soon as possible. The antibiotic regimen will differ from hospital to hospital, so it is best to consult your hospital's infectious diseases department. Cultures should be taken and sent as soon as possible to narrow bacterial sensitivity. Antibiotic treatment, however, should not delay definitive treatment of the infection, which should be aggressive, serial surgical debridement (Fig 2).

Numerous studies have shown a correlation between mortality and the timing and adequacy of the debridement.^3^,^7^ As in the case described, serial debridements (every 24-48 hours) are required. Skin grafting with autograft is a commonly used reconstructive measure if large areas of tissue are involved. Other options for the reconstruction of less extensive tissue destruction would include myocutaneous flaps, regional flaps, and even free flaps.

*Vibrio vulnificus* should be considered particularly when there is exposure of the patient to seawater. It is destructive and difficult to treat, requiring multiple IV antibiotics, commonly doxycycline, a third-generation cephalosporin, and imipenem given at maximal dosage. It usually presents, after 1 day, with swelling, pain, tenderness, ecchymoses, and blistering. Untreated it can rapidly lead to progressive necrotizing fasciitis, sepsis, and death.

## Figures and Tables

**Figure:**
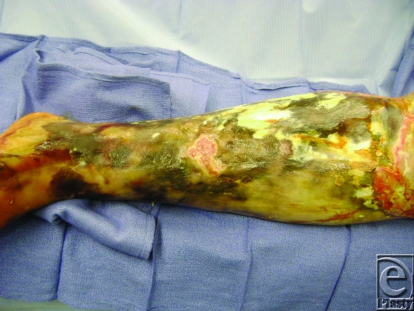


**Figure:**